# Analysis of the Bacterial Diversity of Paipa Cheese (a Traditional Raw Cow’s Milk Cheese from Colombia) by High-Throughput Sequencing

**DOI:** 10.3390/microorganisms8020218

**Published:** 2020-02-06

**Authors:** José Castellanos-Rozo, Rubén Pérez Pulido, Mª. José Grande, Rosario Lucas, Antonio Gálvez

**Affiliations:** 1 Department of Biology and Microbiology, Faculty of Sciences and Engineering, Universidad de Boyacá, 150003 Tunja, Colombia; joscastellanos@uniboyaca.edu.co; 2 Microbiology Division, Department of Health Sciences, Faculty of Experimental Sciences, University of Jaén, 23071 Jaén, Spain; rppulido@ujaen.es (R.P.P.); mjgrande@ujaen.es (M.J.G.); rlucas@ujaen.es (R.L.)

**Keywords:** Paipa cheese, bacterial diversity, lactic acid bacteria, *Staphylococcus*, *Enterobacteriaceae*, *Aromonadaceae*, *Moraxellaceae*

## Abstract

Background: Paipa cheese is a traditional, semi-ripened cheese made from raw cow’s milk in Colombia. The aim of this work was to gain insights on the microbiota of Paipa cheese by using a culture-independent approach. Method: two batches of Paipa cheese from three formal producers were sampled during ripening for 28 days. Total DNA from the cheese samples was used to obtain 16S rRNA gene sequences by using Illumina technology. Results: *Firmicutes* was the main phylum found in the cheeses (relative abundances: 59.2–82.0%), followed by *Proteobacteria*, *Actinobacteria* and *Bacteroidetes*. *Lactococcus* was the main genus, but other lactic acid bacteria (*Enterococcus*, *Leuconostoc* and *Streptococcus*) were also detected. *Stapylococcus* was also relevant in some cheese samples. The most important *Proteobacteria* were *Enterobacteriaceae*, *Aeromonadaceae* and *Moraxellaceae*. *Enterobacter* and *Enterobacteriaceae* (others) were detected in all cheese samples. *Serratia* and *Citrobacter* were detected in some samples. *Aeromonas* and *Acinetobacter* were also relevant. Other minor genera detected were *Marinomonas*, *Corynebacterium* 1 and *Chryseobacterium*. The principal coordinates analysis suggested that there were producer-dependent differences in the microbiota of Paipa cheeses. Conclusions: lactic acid bacteria are the main bacterial group in Paipa cheeses. However, other bacterial groups, including spoilage bacteria, potentially toxin producers, and bacteria potentially pathogenic to humans and/or prone to carry antimicrobial resistance genes are also relevant in the cheeses.

## 1. Introduction

The analysis of microbial communities by culture-dependent methods is laborious and requires the use of selective methods and conditions that are specific for each microbial group. By contrast, culture-independent approaches use more general analytical procedures and also have the advantage of detecting fastidious microorganisms or bacterial cells in the viable but non-culturable state [1,2]. High-throughput sequencing (HTS) has become a powerful culture-independent approach that can be used to obtain snapshots of microbial communities from food systems. In the milk and dairy industry, numerous studies based on HTS technologies have been carried out [3] with different purposes, e.g., to decipher the microbiota from farm environments and animals [4], raw and pasteurized milk [5], transportation tanks, vats, utensils and cheese production facilities [6,7,8] and the variations associated with season [6] or animal feeding [9], to establish relationships between microbial communities in the raw materials, the environment and the finished product [8,9,10], to study the changes in the bacterial communities associated with cheese-making [9,11] and cheese ripening processes [9,12,13,14,15], or to determine the microbial composition of cheeses and cheese varieties [16,17,18,19]. Furthermore, HTS studies can also provide information on adventitious or previously overlooked microbiota or the presence of pathogenic or spoilage bacteria in the cheese and dairy environment [2,3,11,14,20,21].

Paipa cheese is a traditional cheese made from raw cow’s milk by small producers in the municipalities of Paipa and Sotaquirá, Boyacá, Colombia [22,23]. Paipa cheese has received the denomination of protection (DPO) by Colombian regulations [23]. Paipa cheese is produced by ca. 70 microenterprises, of which 15 are formally legal (also known as formal producers) and stamp the sanitary registration and DPO quality seal on their product. Paipa cheese production relies on the artisanal experience of cheesemakers, transmitted from one generation to another. The cheese is made without the addition of starter cultures. After milk collection, the floating cream is removed manually and the milk is coagulated enzymatically. Kneading, pressing and molding are carried out manually according to a traditional process in which the hands, fists and elbows of cheesemakers come in contact with the cheese curd. The cheese is allowed to ripen on wooden shelves at ambient temperatures (which may range between 12 and 20 °C), at a relative humidity of 60-80%. The standard ripening time (21 days) can be extended up to 28 days. The final pH and moisture content of the cheeses should be about 5.2 and 47.4%, respectively. 

Studies on the microbiological aspects of Paipa cheese production process are scarce [22]. Being a traditional process based on raw milk, it is important to understand the microbial composition of the cheese and the variations that may occur in this spontaneous ripening process that relies on the microbiota from the raw material and the processing environment, and to identify possible microbiological risks. The aim of the present study was to gain insights into the bacterial biodiversity of Paipa cheese during the ripening period by using HTS technology. The results obtained indicate that lactic acid bacteria are the main bacterial group found during cheese ripening, but also that other bacterial groups, including potentially spoilage, toxinogenic or pathogenic bacteria can also be present during ripening and in the finished product.

## 2. Materials and Methods

### 2.1. Cheese Sampling

The Paipa cheese was made by local formal producers from raw cow’s milk by enzymatic coagulation and hand molding, following the standard procedure described in the Colombian regulations for the protected denomination of the origin of the cheese [23]. The ripening was done on wooden shelves in rooms under natural conditions (local temperatures of 12–20 °C, relative humidity 60%–70%) with daily turning by hand. The cheese samples were provided by three local cheese producers (C, A, F) in 2018. For each producer, two samplings (A and B) were done on two cheeses from different production batches that were prepared approximately one month apart. The samplings were done at 0, 10, 21 and 28 days of ripening. Briefly, radial slices (ca. 100 g weight each) were separated aseptically from the cheeses with a sterile knife and deposited, under aseptic conditions, inside zip-lock plastic bags. The bags were stored at 4 °C and then transported to the laboratory on ice (<24 h). The samples were stored frozen at −20 °C until the analysis. 

### 2.2. DNA Extraction

The DNA extraction was performed using a PowerFood^™^ Microbial DNA Isolation kit (MoBio Laboratories Inc., Carlsbad, CA, USA) following the instructions provided by the manufacturer for uncultured food. The zip-lock bags containing the cheese samples were left at room temperature for 10 min and then placed inside Stomacher bags. The cheese samples inside the double bags were first broken into small pieces by hand rubbing and then pummeled for 1 min. in a Stomacher 400 (Seward, UK). For each cheese sample, two 0.25 g portions of the obtained homogenate were used in separate extractions and the extracted DNA was pooled into a single test tube. The quality and quantity of the extracted DNA was determined using a QuantiFluor^®^ ONE dsDNA system (Promega, Madison, WI, USA). The DNA was stored at –20 °C until analysis. 

### 2.3. DNA Sequencing and Analysis

The 16S rDNA gene amplicons were obtained following the 16S rDNA gene Metagenomic Sequencing Library Preparation Illumina protocol (Cod. 15044223 Rev. B). The gene-specific sequences used in this protocol target the 16S rDNA gene V3 and V4 region. Illumina adapter overhang nucleotide sequences were added to the gene-specific sequences. The primers were selected from [24]. The following 16S rDNA gene amplicon PCR primer sequences were used: forward primer: 5′TCGTCGGCAGCGTCAGATGTGTATAAGAGACAGCCTACGGGNGGCWGCAG; reverse primer: 5′GTCTCGTGGGCTCGGAGATGTGTATAAGAGACAGGACTACHVGGGTATCTAATCC. Microbial genomic DNA (5 ng/μL in 10 mM Tris pH 8.5) was used to initiate the protocol. After 16S rDNA gene amplification, the multiplexing step was performed using a Nextera XT Index Kit (FC-131-1096). An amount of 1 μL of the PCR product was run on a Bioanalyzer DNA 1000 chip to verify the size (expected size ~550 bp). After size verification, the libraries were sequenced using a 2 × 300 pb paired-end run (MiSeq Reagent kit v3 (MS-102-3001)) on a MiSeq Sequencer, according to manufacturer’s instructions (Illumina, Inc., San Diego, CA., USA). Quality assessment was performed using the prinseq-lite program [25]. The sequence data were analyzed using a qiime2 pipeline [26]. The denoising, paired-ends joining, and chimera depletion were performed, starting from the paired ends data, using a DADA2 pipeline [27]. The taxonomic affiliations were assigned using the Naive Bayesian classifier integrated in the quiime2 plugins and the SILVA_release_132 database [28]. The statistical analysis was carried out with SPSS software version 24 (IBM Corp., Foster City, CA, USA). The sequencing output files will be deposited in the Sequence Read Archive (SRA) service of the European Bioinformatics Institute (EBI) database under Accession Number PRJEB36556.

## 3. Results

### 3.1. Characteristics of Sample Sequence Reads

A total of 49,22,740 reads were obtained from the 24 cheese samples. After the quality control, 3,786,938 merged reads were obtained, of which 3,486,293 were non-chimeric reads. The total number of reads assigned to operational taxonomic units (OTUs) per sample ranged from 100,681 to 189,360 (Table 1). Sample AA10 (sampling A from producer A at day 10 of ripening) was an exception, since it contained a very low number of reads. Furthermore, after OTU assignment, Sample 10 did not show the microbial composition that is typical of cheeses, but instead contained a very high relative abundance of *Pseudomonas*. Therefore, this sample was not included in the analysis after this point. The alpha diversity indices are shown in Table 1.

### 3.2. Bacterial Populations in Cheese Samples

**Figure 1:**
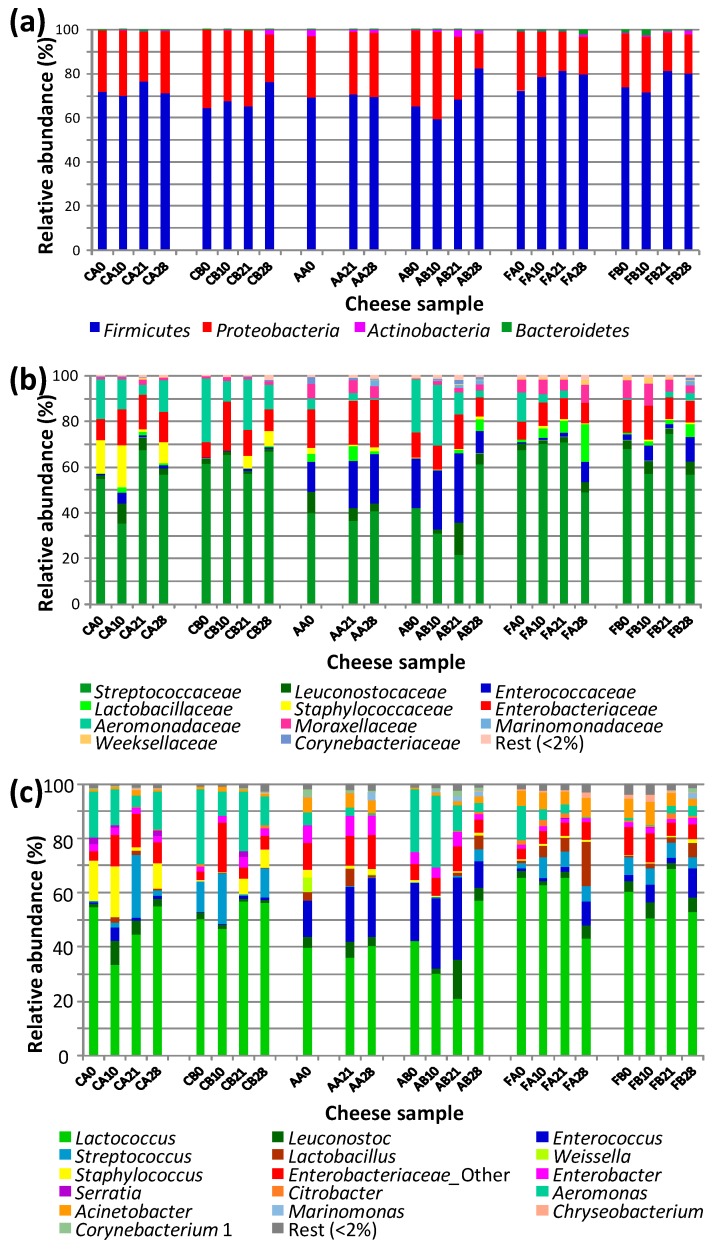
Bacterial diversity of Paipa cheese samples at phylum (**a**), family (**b**) and genus (**c**) levels. Cheese samples from three formal producers (C, A, F) corresponding to two independent batches (A and B) at different ripening times (0, 10, 21, 28 days) were analyzed.

*Firmicutes* was the main bacterial phylum found in the cheeses, with relative abundances ranging from 59.2% up to 82% (Figure 1a). The second main phylum was *Proteobacteria*, with relative abundances ranging from 15.93% to 39.50%. *Actinobacteria* and *Bacteroidetes* had much lower relative abundances, always below 3.5%. *Firmicutes* were represented mainly by members of O. *Lactobacillales*. The OTUs with the highest relative abundances in most of the cheese samples (from 21.45% to 74.40%) belonged to Fam. *Streptococcaceae* (Figure 1B). The main genus detected in this group was *Lactococcus* (Figure 1C), which was detected in all cheese samples (at relative abundances ranging between 20.96% and 68.64%). The second group in relative abundance was Fam. *Enterococcaceae*, with the genus *Enterococcus* as the main representative. The genus *Enterococcus* was the most prevalent in the cheeses from producer A compared to the other cheeses, reaching up to 30.38% in one sample. *Leuconostoc* and *Streptococcus* were also found in many other cheese samples, although they had much lower relative abundances in general. The genus *Staphylococcus* was detected at relative abundances ≥2% in 6 samples (all of them belonging to cheeses from producers C and A), reaching 18.63% in one sample.

*Proteobacteria* were represented mainly by *Gammaproteobacteria* belonging to Fam. *Enterobacteriaceae* and *Aeromonadaceae* and, to a lesser extent, also *Moraxellaceae* (Figure 1b). Fam. *Enterobacteriaceae* was detected in all cheese samples, with relative abundances comprised between 3.36% and 12.30%. The OTUs assigned to the genus *Enterobacter* were also detected in all cheese samples, although they had lower relative abundances (Figure 1c). *Serratia* was detected at relative abundances between 2% and 3% in three of the C cheese samples. *Citrobacter* was also relevant, especially in samples from producer F (reaching 2.30% in one sample). Within Fam. *Aeromonadaceae*, the genus *Aeromonas* was an important OTU in most cheese samples, reaching high relative abundances in some cases (up to 27.60%). *Acinetobacter* (Fam. *Moraxellaceae*) was also detected frequently at relative abundances of up to 8.60%, mainly in the FA and AB cheese samples. Other genera (*Marinomonas*, *Corynebacterium* 1 and *Chryseobacterium*) were only detected at relative abundances ≥ 2% in some cheese samples. 

No major changes in the bacterial populations were observed during ripening. The relative abundance of *Lactococcus* only increased slightly but non-significantly (*p* > 0.05) by the end of ripening in cheese samples CA (day 21), CB (days 21 and 28) and FB (day 21) but in some cases decreased (AB21, FA28). 

**Figure 2:**
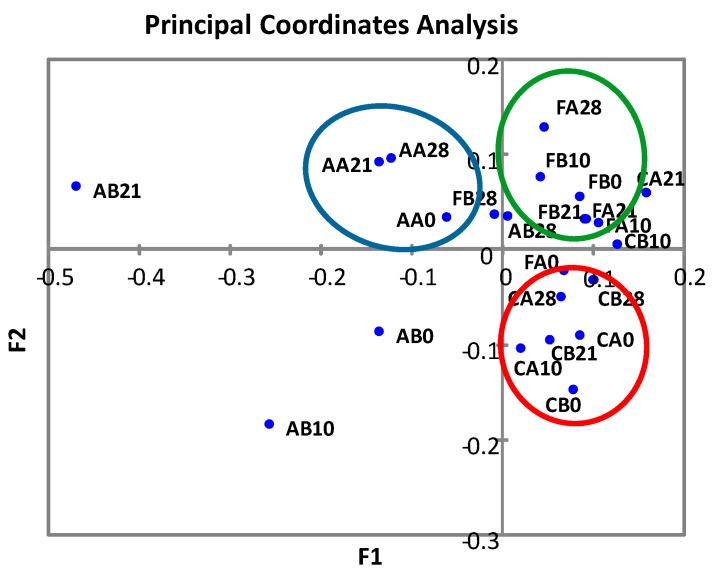
Principal coordinates analysis of Paipa cheese microbial composition. Cheese samples from three formal producers (C, A, F) corresponding to two independent batches (A and B) at different ripening times (0, 10, 21, 28 days) were analyzed. The ellipses indicate the clustering of the samples from producers C (red), A (blue) and F (green).

The principal coordinates analysis (PcoA) revealed that most of the cheese samples clustered according to the sample origin (local producer) and independently of the production batch (Figure 2). However, there were also clear outliers, especially in the samples from cheese producer A. A hallmark of the cheeses from producer A was the higher relative abundance of OTUs assigned to the genus *Enterococcus*.

## 4. Discussion

The results from the present study based on culture-independent high-throughput amplicon sequencing technology provided insights into the microbiota of Paipa cheese produced by traditional methods in formal enterprises. The differences in the microbial composition of the cheeses found between the three producers studied suggest that resident microbiota and different hygiene practices contribute to the final microbiota of the cheeses. Nevertheless, we should remark that amplicon sequencing only provided a resolution at genus level, which may be insufficient for those genera that comprise pathogenic species or are of technological relevance to the cheese-making process. Therefore, the identification at genus level provides only general hints as to the actual role of the bacterial populations present in the studied matrix. Further studies based on culture-dependent methods should be carried out in order to accomplish species/strain identification.

The results of the amplicon sequence analysis could also be influenced by the DNA extraction method. A previous study comparing different DNA extraction methods on raw milk cheese concluded that the MoBio PowerFood^™^ Microbial DNA Isolation kit performed the best compared to other DNA extraction methods for the recovery of highly concentrated and pure DNA that could then be used for the accurate detection of foodborne pathogens by quantitative PCR [29]. The same DNA extraction kit was reported to provide satisfactory results in microbial community studies carried out on milks [5,6,30,31] and experimental cheese [31].

As is to be expected for a fermented dairy product, *Firmicutes* was the main bacterial group found in all cheese samples from the three producers. Members of the genus *Lactococcus* seem to be the main actors of lactic acid fermentation in Paipa cheese, as shown by the high relative abundance of OTUs corresponding to this group in all cheese samples. Other genera of lactic acid bacteria, like *Leuconostoc*, *Streptococcus*, *Enterococcus* or *Lactobacillus* were also detected, but they showed large variations in their relative abundances between samples. 

Particularly, OTUs assigned to the genus *Enterococcus* were detected in all cheese samples and showed the highest relative abundances in samples from producer A, while samples from producer F showed the lowest. Enterococci can be found in many different habitats, such as soil, water, foods, and the gastrointestinal tract of animals. The presence of enterococci in foods is a controversial issue. Enterococci are commonly found in cheeses made from raw or pasteurized milk, where they can play several beneficial roles in cheese fermentation and ripening [32]. Due to their proteolytic, esterolytic and lipolytic activities, citrate breakdown and production of diacetyl, and other important volatile compounds, enterococci participate in the development of the cheese-specific flavor, taste and texture [32,33,34]. Furthermore, many enterococci may also produce bacteriocins with antimicrobial activity against foodborne pathogens that may occur in cheese, such as *Listeria monocytogenes* and *Staphylococcus aureus* [35,36]. Therefore, the technological potential of enterococci from Paipa cheeses deserves further investigation. Enterococci may also have a negative impact in foods (including cheeses) through the production of biogenic amines [36,37]. There is a concern about the presence of enterococci in foods, since these bacteria may also behave as opportunistic pathogens and cause different infections in humans [36,38]. Enterococci are also worrisome because of their ability to acquire and transfer antibiotic resistance traits [38,39,40,41]. Therefore, further studies should be carried out to determine the safety of enterococci from Paipa cheeses, specially the potential for production of biogenic amines and the presence of virulence factors and antibiotic resistance traits.

Many cheese samples yielded high relative abundances of OTUs assigned to the genus *Staphylococcus*, especially the samples from producer C. In contrast, the relative abundances in most samples from producer A and in all samples from producer F were very low. The main sources of staphylococci in the cheese could be the udders of lactating cows and the hands of cheese workers [42]. The traditional practice of molding by hand and the daily turning of Paipa cheese manually could explain the presence of staphylococci in the cheeses. The observed differences between the producers regarding staphylococci could be attributed to differences in hygienic practices. The presence of members of the genus *Staphylococcus* in Paipa cheese should be a matter of concern, since this bacterial group may contain species or strains that may cause human infections or produce enterotoxins. *S. aureus* is the main enterotoxin producer within the genus. *S. aureus* can grow in milk and produce heat-stable enterotoxins. The growth of *S. aureus* in cheeses and the production of enterotoxins are influenced by environmental conditions, such as temperature, pH, water activity, salt concentration, and bacterial competition [43]. There is also a concern about the presence of antibiotic-resistant staphylococci in cheeses. Recent studies reported the presence of methicillin-resistant *S. aureus* (MRSA) in Doble Crema cheese (a fresh cheese similar to mozzarella and other stretched curd cheeses, traditionally made from raw cow’s milk in small dairies in Colombia) [44] and in artisanal cheeses from Mexico [45], Brazil [46] and Egypt [47], among others. Furthermore, cheeses may also contain species of coagulase-negative *Staphylococcus* and these bacteria may carry antimicrobial resistance and enterotoxin production traits as well [48,49,50,51].

All cheese samples yielded high percentages of OTUs belonging to *Proteobacteria*. Most members of this group found in the cheese included spoilage bacteria, but also some important human pathogens. Fam. *Enterobacteriaceae* was one the two main bacterial groups within *Proteobacteria* in Paipa cheese. Most of the OTUs detected in cheese samples belonged to the genus *Enterobacter* or could only be assigned to Fam. *Enterobacteriaceae* (others), and these were present in all cheese samples. These results suggest the presence of spoiling *Enterobacteriaceae* in the cheeses, most likely coming from the raw milk used for cheese making. Nevertheless, potentially human-pathogenic members of *Enterobacteriaceae*, like *Serratia* (which was relevant in three samples from producer C) and *Citrobacter* (which was relevant in some samples from producer F) were also detected. These results could be an indication of the health risks associated with the consumption of Paipa cheese.

The genus *Aeromonas* was also highly represented in the Paipa cheese samples. The OTUs assigned to this bacterial group showed the highest relative abundances at the beginning of the ripening period (especially in cheese samples from producer C and also in one of the batches from producers A and F). The presence of *Aeromonas* in milk and dairy products has been reported in several culture-dependent studies, e.g., in raw herd’s milk samples from Switzerland [48], in raw cow’s milk, local plain yoghurt and Domiati cheese from Egypt [52], in Anthotyros and Manouri Greek cheeses [53], in Turkish Urfa cheese [54] and in Spanish Villalón cheese [55]. *Aeromonas* spp. has also been reported in several studies on milk and fresh cheeses from Brazil [56,57]. In Minas Frescal cheese, *Aeromonas* spp. was isolated in raw and pasteurized milk, in the environment and on the handlers’ hands. During the processing of Colonial cheese, *Aeromonas* spp. was isolated in the water, raw milk, after thermal treatment and curd, as well as on the handlers’ hands and utensils [58]. *Aeromonas* spp. are important emerging microorganisms in foodborne diseases [59,60]. *Aeromonas* spp. may cause human illnesses, ranging from gastroenteritis to invasive disease. The most common cause of *Aeromonas* infection is the ingestion of contaminated water, but transmission may also occur through contaminated foods [59,60]. According to the results from the present study, *Aeromonas* should be considered a bacterium of concern for the microbiological safety of Paipa cheese.

The OTUs assigned to the genus *Acinetobacter* were also detected in all cheese samples, showing the highest relative abundances in samples from producers A and F. *Acinetobacter* species are ubiquitous in soil, water and different types of foods, including milk and cheese, being often associated with spoilage [61]. One study reported that acinetobacters isolated during the maturation of Camembert cheese showed lipolytic actvity and were able to utilize citrate, two properties that could explain their ability to grow in the cheese ecosystem [62]. Another study reported that *Acinetobacter* isolates from mozzarella cheese were proteolytic [63]. *Acinetobacter* has been detected by high-throughput sequencing in several studies, including raw bovine milk from tanker trucks and silos [6], vats and other environmental samples [7], milk, curd and Pico cheese (an artisanal cheese manufactured from raw cow’s milk in small dairy units in Portugal) [11,14] and in Chinese Rushan cheese [18]. The presence of *Acinetobacter* in Paipa cheese could be important not only as an indication of poor hygiene and environmental contamination, but also because members of this group may also carry antibiotic resistance genes.

Compared to conventional cheeses (where heat treatment of the milk and the rapid acidification produced by added starter cultures minimize the survival and proliferation of potentially pathogenic bacteria), artisanal cheeses, like Paipa cheese, rely on natural fermentation where lactic acid bacteria does not always succeed in displacing other microbial populations that may pose risks to human health or may cause spoilage [44,45,46,47]. There is a delicate balance between preserving the unique flavors of artisanal cheeses and ensuring cheese safety.

## 5. Conclusions

In conclusion, the results from the present study revealed that the core microbiota of Paipa cheeses not only include lactic acid bacteria, but also spoilage bacteria, potentially enterotoxin producers, and human pathogens. This happens despite the fact that all three suppliers of the cheese samples were formal producers, which suggests a need for an improvement or reinforcement in the regulations for the production of traditional Paipa cheese.

## Figures and Tables

**Table 1:** N° of reads and alpha diversity indexes at the genus level of the Paipa cheese samples.

Sample	N°Reads	N°Observations	Chao1	ACE	Shannon	Simpson
CA0	119123.00	32.00	32.00	32.00	1.50	0.65
CA10	117789.00	28.00	28.00	28.00	1.98	0.81
CA21	100681.00	33.00	33.00	33.00	1.73	0.73
CA28	106138.00	32.00	32.00	32.00	1.64	0.66
CB0	101205.00	25.00	25.00	25.00	1.41	0.66
CB10	105216.00	27.00	27.00	27.00	1.55	0.71
CB21	112479.00	32.00	32.00	32.00	1.45	0.62
CB28	104831.00	31.00	31.00	31.00	1.65	0.66
AA0	124879.00	33.00	33.00	34.08	2.10	0.80
AA10	6744.00	13.00	13.00	13.00	1.71	0.67
AA21	130445.00	30.00	30.00	30.00	1.99	0.80
AA28	125251.00	35.00	35.00	35.00	1.89	0.77
AB0	189360.00	26.00	26.00	26.00	1.52	0.72
AB10	153619.00	31.00	31.00	31.35	1.74	0.77
AB21	158773.00	34.00	34.00	34.44	2.05	0.82
AB28	157004.00	41.00	41.00	41.00	1.74	0.66
FA0	161174.00	41.00	41.00	41.00	1.40	0.55
FA10	128947.00	37.00	37.00	37.00	1.57	0.59
FA21	151273.00	39.00	39.00	39.00	1.50	0.56
FA28	151121.00	45.00	45.00	45.00	1.91	0.76
FB0	152092.00	44.00	44.00	44.00	1.59	0.61
FB10	162325.00	51.00	51.00	51.00	1.86	0.71
FB21	177231.00	45.00	45.00	45.00	1.41	0.52
FB28	160890.00	44.00	44.00	44.00	1.86	0.70
